# Clinical Characteristics of Asymptomatic Patients with SARS-CoV-2 in Zhejiang: An Imperceptible Source of Infection

**DOI:** 10.1155/2020/2045341

**Published:** 2020-09-19

**Authors:** Wei Dai, Xinmiao Chen, Xiaoting Xu, Zhefeng Leng, Wenwen Yu, Hui Lin, Huiying Li, Jie Lin, Zhangwei Qiu, Yuanrong Dai

**Affiliations:** ^1^Department of Neurorehabilitation, The Second Affiliated Hospital of Wenzhou Medical University, Wenzhou 325027, China; ^2^Department of Respiratory and Critical Care Medicine, The Second Affiliated Hospital of Wenzhou Medical University, Wenzhou 325027, China

## Abstract

**Objective:**

Coronavirus disease 2019 (COVID-19), caused by the novel coronavirus SARS-CoV-2, was first identified in December 2019 in Wuhan, China, and has since spread globally, resulting in an ongoing pandemic. However, the study of asymptomatic patients is still rare, and the understanding of its potential transmission risk is still insufficient. In this study, epidemiological investigations were conducted in the Zhejiang province to understand the epidemiology and clinical characteristics of asymptomatic patients with COVID-19.

**Methods:**

This retrospective study was carried out on 22 asymptomatic patients and 234 symptomatic patients with COVID-19 who were hospitalized in Zhejiang Duodi Hospital from January 21 to March 16, 2020. The characteristics of epidemiology, demography, clinical manifestations, and laboratory data of mild patients were compared and analyzed.

**Results:**

The median age was 28 years in asymptomatic patients and 48 years in symptomatic patients. The proportion who were female was 77.3% in asymptomatic patients and 36.3% in symptomatic patients (*p* < 0.001). The proportion of patients with coexisting diseases was 4.5% in asymptomatic patients and 38.0% in symptomatic patients (*p*=0.002). The proportion of patients with increased CRP was 13.6% in the asymptomatic group and 61.1% in the symptomatic group (*p* < 0.001). The proportion of patients received antiviral therapy was 45.5% in the asymptomatic group and 97.9% in the symptomatic group (*p* < 0.001). The proportion of patients received oxygen therapy was 22.7% in the asymptomatic group and 99.1% in symptomatic patients (*p* < 0.001). By March 16, 2020, all patients were discharged from the hospital, and no symptoms had appeared in the asymptomatic patients during hospitalization. The median course of infection to discharge was 21.5 days in asymptomatic patients and 22 days in symptomatic patients.

**Conclusions:**

Asymptomatic patients are also infectious; relying only on clinical symptoms, blood cell tests, and radiology examination will lead to misdiagnosis of most patients, leading to the spread of the virus. Investigation of medical history is the best strategy for screening asymptomatic patients, especially young people, women, and people without coexisting disease, who are more likely to be asymptomatic when infected. Although the prognosis is good, isolation is critical for asymptomatic patients, and it is important not to end isolation early before a nucleic acid test turns negative.

## 1. Introduction

Coronavirus disease 2019 (COVID-19) is caused by the beta-coronavirus SARS-CoV-2 [1]. It was first identified in Wuhan and soon became an epidemic, spreading rapidly, and is still not fully controlled to this day. The scope of the epidemic [2–5] has drawn great attention from scholars from various countries, and much research has been done on its epidemiology, clinical characteristics, diagnosis, and treatment measures [2, 6–9]. Some patients with COVID-19 are asymptomatic and show no significant signs on chest CT. Although the number of confirmed asymptomatic cases is small, the actual patient population may be quite high [10], they are likely to become potential sources of infection, and, at the same time, their conditions may worsen and then develop into the ordinary symptomatic type. Though very little attention is paid to asymptomatic patients, a study on asymptomatic patients had shown that asymptomatic patients are infectious [11]. This study included 22 cases who were confirmed as asymptomatic COVID-19 and were selected by medical institutions in multiple counties and cities in the Zhejiang province in order to explore its epidemiological and clinical characteristics.

## 2. Research Objectives and Methods

### 2.1. Research Objective

This retrospective study included a total of 256 laboratory-confirmed COVID-19 patients admitted to various medical institutions in Zhejiang from January 21 to March 16, 2020, 22 of whom were asymptomatic. All patients were diagnosed on the basis of the World Health Organization interim guidance [12]. Real-time reverse transcription polymerase chain reaction (RT-PCR) was performed to screen the patients' sputum, throat swab specimen, or feces. The RT-PCR detection reagent was provided by Shanghai Biomedical Biotechnology Co., Ltd. The primer sequences were forward primer 5′-CCCTGTGGTTTACTAA-3′; reverse primer 5′-ACGATTGCATCACTGA-3′. The amplification conditions were 50°C for 15 minutes, 95°C for 3 minutes, and then 45 cycles of 95°C for 15 seconds and 60°C for 30 seconds. This study was approved by the Ethics Committee of the Second Affiliated Hospital of Wenzhou Medical University and Yuying Children's Hospital (L-2020-13).

### 2.2. Research Methods

We extracted epidemiological, demographic, clinical, laboratory, radiological, and therapeutic data from patient records and grouped patients into symptomatic and asymptomatic groups according to their clinical symptoms including fever, cough, nasal congestion, diarrhea, muscle soreness, pharyngalgia, and chest tightness. The laboratory data included complete blood counts, C-reactive protein (CRP), procalcitonin (PCT), D-dimer, liver and kidney function, and blood chemistry. All patients underwent a computed tomography (CT) scan of the chest. Toshiba Aquilion 64-row spiral CT was used by professional imaging doctors to scan the patients from the apex to the bottom of the lung with a layer thickness of 1.0 mm and a scan time of 5-6 s.

### 2.3. Statistical Methods

For continuous random variables, the mean and standard deviation (SD) were selected. Data were analyzed by using independent sample *t*-tests. The difference was statistically significant if *p* < 0.05. Categorical variables were summarized as counts and percentages for each category and expressed as a percentage of numbers, and the asymptomatic group was compared with the symptomatic group by the *χ*^2^ test, continuous correction *χ*^2^ test, or Fisher exact test. Statistical analysis was performed by using SPSS software, version 21.0.

## 3. Results

### 3.1. Demographic Data

We included 22 hospitalized patients who were confirmed as asymptomatic COVID-19 after investigating their medical history with exposure risk to prevent the progression and as a quarantine measure, accounting for 8.6% of the total collected cases (234 in total). No patients had been exposed to wildlife. 10 (45.5%) asymptomatic patients had visited Wuhan or had been in (direct or indirect) contact with people from Wuhan. There was one patient with clustered infection in the family. Five others had family members who were diagnosed with COVID-19 pneumonia, and all of them denied contact with relevant people from Wuhan.

### 3.2. Basic Features

In the 22 asymptomatic patients, 5 (22.7%) were male and 17 (77.3%) were female, but of the 234 symptomatic patients, 73 (62.4%) were male and 44 (37.6%) were female. The difference was significant (*p* < 0.001) (Table 1).

**Figure 1:**
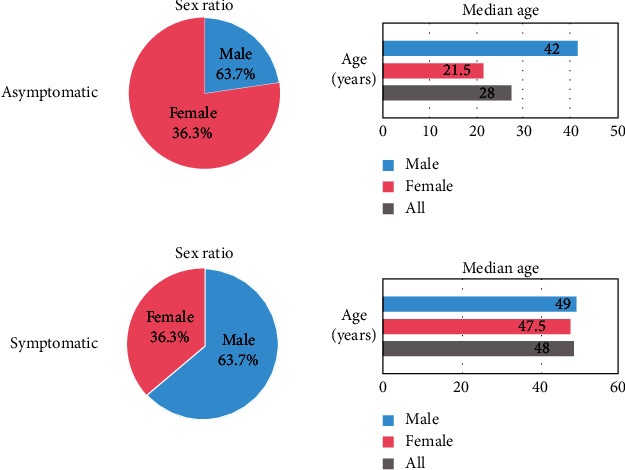
Basic features. The sex ratio is shown in the pie chart on the left, and the median age comparison of different genders is shown in the figure on the right. The asymptomatic patients and the symptomatic patients are compared, respectively.

The median age of asymptomatic patients was 28 years (range: 10–68), with a median age of 21.5 years for women and 42 years for men. The median age of symptomatic patients was 48 years (range: 20–77), with a median age of 47.5 years for women and 49 years for men (Figure 1). The mean age of asymptomatic patients was significantly lower than that of symptomatic patients (33.0 years versus 47.6 years, *p* < 0.001).

Among the asymptomatic patients, only 1 (4.5%) patient had diabetes as a coexisting disease, while in the symptomatic group, 40 (38.0%) patients had coexisting disease, a difference which was statistically significant (Table 1). None of the asymptomatic patients had a history of smoking.

### 3.3. Clinical Characteristics

#### 3.3.1. Clinical Symptoms

Though no symptoms showed in the asymptomatic group, fever (80.8%) and cough (73.5%) were common in symptomatic patients, and nasal congestion (6.4%) was a relatively rare symptom (Table 2).

#### 3.3.2. Laboratory Findings

All patients had blood tests. Patients with a rise in CRP were significantly fewer in the asymptomatic group (13.6% versus 61.1%, *p* < 0.001). The proportion of patients with leukopenia was almost the same between two groups (22.7% versus 22.2%, *p* > 0.999). The patients with decreased lymphopenia was 13.6% in the asymptomatic group and 26.5% in the other, which was not statistically significant (*p*=0.185) (Table 3).

In the asymptomatic group, other than 1 (4.5%) patient with lymphocytosis, 1 (4.5%) with neutrophilia, and 1 (4.5%) with thrombocytosis, no eosinophil count, D-dimer, alanine aminotransferase (ALT), PCT, total bilirubin (TBil), urea nitrogen (BUN), or serum creatinine (Cr) abnormalities were found.

#### 3.3.3. Radiology

The difference in radiology between the two groups was obvious. In the symptomatic group, 216 (92.3%) patients had different degrees of chest CT abnormalities, and only 2 (9.1%) patients in the asymptomatic group were suggested to have pneumonia by radiology (*p* < 0.001) (Table 4).

### 3.4. Treatment

In the symptomatic group, 229 (97.9%) patients received antiviral treatment and 232 (99.1%) patients received oxygen therapy, while in the asymptomatic group, 10 (45.5%) received antiviral treatment and 5 (22.7%) received oxygen therapy, which were statistically different (both *p* < 0.001). 12 (5.1%) patients received glucocorticoid therapy in the symptomatic group, no patients received the therapy in the asymptomatic group, and no statistical difference was found between the two groups (*p*=0.575) (Table 5).

### 3.5. Treatment Results

**Figure 2:**
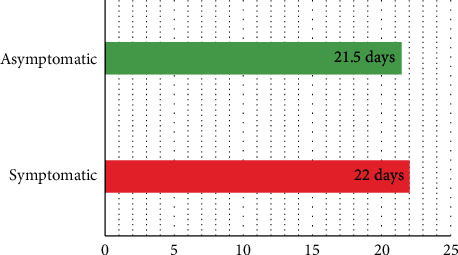
Median course of disease. The median course of asymptomatic and symptomatic patients was compared intuitively in the bar graph.

By March 16, 2020, all patients were discharged from the hospital, and no symptoms appeared in any asymptomatic patients during hospitalization. The median course of disease (from symptom onset to discharge for symptomatic patients and from diagnosis to discharge for asymptomatic patients) was 22 (10–36) days in the symptomatic group and 21.5 (10–34) days in the discharged asymptomatic group (Figure 2).

## 4. Discussion

In our study, 22 confirmed asymptomatic COVID-19 inpatients were included from multiple regions in Zhejiang, accounting for 8.6% of all cases collected. However, because all these cases are confirmed after medical history investigation, the number is likely to be much lower than the actual number, and many other studies have made similar assumptions [13–17]. The reasons are discussed below.

In asymptomatic patients, CRP increase was less prevalent, which was relatively characteristic in symptomatic patients [13–15]. The abnormality of leukocyte count was not seen, and the results of other blood cell tests were mostly normal. Besides, the proportion of chest CT abnormalities in asymptomatic patients was also very low. Therefore, it is difficult to diagnose asymptomatic patients only by the results of blood cell tests and radiology examination, which may cause an increase in missed diagnosis rate.

Though without symptoms, asymptomatic patients are no less infectious than the symptomatic patients [18].

In addition, 42.2% of all patients have no obvious contact history. Though the investigation is difficult, asymptomatic patients may play an important role in the infection of these patients. It is possible that even the asymptomatic patients who have already recovered do not know that they have been infected, but become a virus carrier [16].

There are three obvious basic characteristics in the asymptomatic group. First, the asymptomatic group was significantly younger, and the patient of a family cluster case aged 10 years was the only one in the family without symptoms. Second, the proportion of female patients in the asymptomatic group was significantly higher. Third, the proportion of asymptomatic patients with coexisting diseases was significantly lower. It can be concluded that those who are young, female, and without coexisting diseases are more likely to be asymptomatic.

For the diagnosed asymptomatic patients, their treatment is relatively little and simple, and their prognosis is obviously better [13–15], but their duration of infection is no different from that of the symptomatic patients. For asymptomatic patients, the onset of the course of disease is the time of diagnosis so the course of asymptomatic patients may be longer [19]. So as a source of infection, isolation is relatively more important for the asymptomatic patients [18].

To sum up, asymptomatic patients are likely to be infectious. The absence of clinical symptoms, normal blood cell examination, and radiology examination causes a high rate of missed diagnosis and underestimation of its proportion, making these patients more of an infection risk. For prevention and control of the spread of virus, especially the entry of air routes, since COVID-19 has become a global pandemic [20], the investigation of history with risk exposure is the main screening method for asymptomatic patients, especially for youth, women, and people without coexisting diseases who are more likely to be asymptomatic when infected, and the nucleic acid test should be carried out on suspected cases. The prognosis of asymptomatic patients is relatively good, but they still need to be isolated for observation as a source of infection. It is unreasonable to shorten the treatment or isolation time as the disease course of asymptomatic patients is not shorter than that of symptomatic patients. The result of the nucleic acid test should be taken as the evidence of cure instead of symptoms to avoid the spread of the disease [12, 18].

## Figures and Tables

**Table 1:** Basic features.

	Asymptomatic (*n* = 22)	Symptomatic (*n* = 234)	*χ* ^2^ value	*t* value	*p* value
Median age (years)	28				
10–68	48				
Age range (years)		20–77			
Mean age (years)	33.0	47.6		−5.134	<0.001
Female patients	17 (77.3%)	85 (36.3%)	14.068		<0.001
Patients with coexisting disease	1 (4.5%)	89 (38.0%)	9.893		0.002

**Table 2:** Clinical symptoms.

	Asymptomatic (*n* = 22)	Symptomatic (*n* = 234)
Fever	0 (0%)	189 (80.8%)
Cough	0 (0%)	172 (73.5%)
Nasal congestion	0 (0%)	15 (6.4%)

**Table 3:** Laboratory tests.

	Asymptomatic (*n* = 22)	Symptomatic (*n* = 234)	*χ* ^2^ value	*p* value
Leukocyte count < 4 × 10^9^/L	5 (22.7%)	52 (22.2%)	0.000	>0.999
CRP > 10 mg/L	3 (13.6%)	143 (61.1%)	18.495	<0.001
Lymphocyte count < 1.0 × 10^9^/L	3 (13.6%)	62 (26.5%)	1.755	0.185

**Table 4:** Radiology.

	Asymptomatic (*n* = 22)	Symptomatic (*n* = 234)	*χ* ^2^ value	*p* value
CT abnormal	2 (9.1%)	216 (92.3%)	103.684	<0.001

**Table 5:** Therapy.

	Asymptomatic (*n* = 22)	Symptomatic (*n* = 234)	*χ* ^2^ value	*p* value
Antiviral therapy	10 (45.5%)	229 (97.9%)	80.839	<0.001
Oxygen therapy	5 (22.7%)	232 (99.1%)	159.969	<0.001
Glucocorticoid therapy	0 (0%)	12 (5.1%)	0.314	0.575

## Data Availability

The nature of the data is epidemiological, demographical, clinical manifestation, and laboratory data of 22 asymptomatic patients and 234 symptomatic patients with COVID-19 who were hospitalized in Zhejiang Duodi Hospital from January 21 to March 16, 2020. The data cannot be accessed because the hospital has the obligation to protect the privacy of patients' hospitalization information.
