# Corrigendum to “Clinical Characteristics of Asymptomatic Patients with SARS-CoV-2 in Zhejiang: An Imperceptible Source of Infection”

**DOI:** 10.1155/2023/9784697

**Published:** 2023-02-07

**Authors:** Wei Dai, Xinmiao Chen, Xiaoting Xu, Zhefeng Leng, Wenwen Yu, Hui Lin, Huiying Li, Jie Lin, Zhangwei Qiu, Yuanrong Dai

**Affiliations:** ^1^Department of Neurorehabilitation, The Second Affiliated Hospital of Wenzhou Medical University, Wenzhou 325027, China; ^2^Department of Respiratory and Critical Care Medicine, The Second Affiliated Hospital of Wenzhou Medical University, Wenzhou 325027, China

In the article titled “Clinical Characteristics of Asymptomatic Patients with SARS-CoV-2 in Zhejiang: An Imperceptible Source of Infection” [1], there is an error in Section 3.2, Basic Features.

“In the 22 asymptomatic patients, 5 (22.7%) were male and 17 (77.3%) were female, but of the 234 symptomatic patients, 73 (62.4%) were male and 44 (37.6%) were female. The difference was significant (*p* < 0.001) (Table 1)” should be corrected to “In the 22 asymptomatic patients, 5 (22.7%) were male and 17 (77.3%) were female, but of the 234 symptomatic patients, 149 (63.7%) were male and 85 (36.3%) were female. The difference was significant (*p* < 0.001) (Table 1).”
